# Awareness of HCV Status and Preferences for Testing and Treatment among People with Recent Injecting Drug Use at a Peer-Led Needle and Syringe Program: The TEMPO Pilot Study

**DOI:** 10.3390/v14112463

**Published:** 2022-11-07

**Authors:** Anna Conway, Phillip Read, Rosie Gilliver, Tony McNaughton, Heather Valerio, Evan B. Cunningham, Charles Henderson, Brett Hadlow, Katrina Molloy, Anna Doab, Shane Tillakeratne, Lucy Pepolim, Mary Ellen Harrod, Gregory J. Dore, Jason Grebely

**Affiliations:** 1 The Kirby Institute, UNSW Sydney, Sydney 2052, Australia; 2 Centre for Social Research in Health, UNSW Sydney, Sydney 2052, Australia; 3 Kirketon Road Centre, South Eastern Sydney Local Health District, Sydney 2010, Australia; 4 NSW Users and AIDS Association, Sydney 2010, Australia

**Keywords:** hepatitis C, testing, treatment, PWID, drug use, injecting drug users, DAA

## Abstract

Background: New technologies and therapies allow the possibility of a single-visit test and treat model for hepatitis C virus (HCV), addressing some of the barriers to care faced by people who inject drugs. Methods: The TEMPO Pilot Study was an interventional cohort study evaluating a single-visit test and treat intervention among people with recent injecting drug use at a one peer-led needle and syringe program (NSP) in Sydney, Australia between September 2019 and February 2021. This analysis evaluated awareness of HCV status and agreement of self-report with HCV RNA test results. The analysis also assessed acceptability of: modality of result delivery, modality of blood sampling, site of treatment, and duration of treatment. Results: Among 101 participants (median age 43; 31% female), 100 had a valid HCV RNA test result and 27% (27/100) were HCV RNA detectable. Overall, 65% (65/100) were aware of their status. Among people with a positive HCV RNA result, 48% (13/27) were aware of their status. People preferred same-day HCV test results (95%, 96/101), and preferred to receive results in person (69%, 70/101). Receiving treatment at an NSP was acceptable (100%, 101/101) and 78% (79/101) were willing to discuss their health with a peer NSP worker. Conclusion: Half of people with current HCV infection were aware of their status. The high acceptability of simplified testing and treatment pathways delivered at NSPs indicates that this is an appropriate strategy to improve HCV awareness and treatment uptake in this population.

## 1. Introduction

The World Health Organization has set a goal to eliminate hepatitis C virus (HCV) infection as a major public health threat by 2030, with targets to increase hepatitis C virus (HCV) diagnoses and treatment, and reduce new infections and liver-related deaths [1]. One barrier to increasing uptake of HCV testing and treatment is the requirement of multiple visits in most current diagnostic pathways, resulting in frequent loss to follow-up [2] which is amplified in key populations such as people who inject drugs [3]. Ensuring that people who inject drugs know their HCV status is an important step towards achieving HCV elimination. Once people are tested and know their status, they are empowered to make decisions about their own health including initiating treatment. People who know their status are better informed to adapt behaviours related to HCV transmission [4].

Same day test and treat models have achieved high proportions of treatment initiation [5,6,7,8]. By delivering results quickly, often outside of traditional healthcare settings, point-of-care testing for HCV RNA can improve access to testing for underserved populations such as people who inject drugs [5,9], homeless people [10], and people that are incarcerated [11]. There has been investigation into acceptability of fingerstick testing [12] but little research on acceptability and preferences for the other, numerous aspects of testing and treatment provision. Co-locating HCV care in services used by people who inject drugs has demonstrated higher treatment uptake and cure [13,14,15,16]. Studies have shown that embedding HCV care in needle syringe programs (NSPs) is acceptable for people who inject drugs [17]. More investigation is needed to understand the acceptability of assays used in same visit HCV RNA testing and treatment in NSPs.

This study presents the awareness of HCV status and acceptability of different modalities of HCV testing and treatment among a sample of people who inject drugs attending a peer-led NSP.

## 2. Methods

### 2.1. Study Design and Participants

In this single centre interventional cohort study, we enrolled participants from one peer-led NSP site in Sydney, Australia from September 2019 and February 2021 (TEMPO Pilot Study, ClinicalTrials.gov: NCT02940691). Study recruitment was halted due to COVID-19 between March-August 2020.

Participants were 18 years or older and had recently injected drugs (self-reported injecting drug use within the last month of enrolment).

### 2.2. Procedures

The TEMPO Pilot Study was advertised preceding recruitment via posters, cards distributed with injecting equipment, and word of mouth through interactions with staff at the NSP site.

Assessments at enrolment included test for HCV RNA, FibroScan transient elastography (FibroScan^®^, Echosens, Paris, France) performed by a specialist nurse, and peer-administered behavioural questionnaires on tablet computers (demographics, behavioural risk, and HCV history data). FibroScan result was provided to the participant in the same visit to discuss with the specialist nurse. The peer worker responsible for the study was an HCV specialist. All participants were compensated (AUD$30 cash).

### 2.3. Laboratory Data

Current HCV infection was assessed by testing 100μL finger-stick capillary whole-blood samples from using the point-of-care Xpert HCV Viral Load Fingerstick assay (Cepheid, Sunnyvale, CA, United States, lower limit of quantification 100 IU/mL; upper limit of quantification 108 log10 IU/mL). When compared to venous blood samples, this assay has a sensitivity and specificity for HCV RNA detection of 99% (95% confidence interval [CI], 97–99%) and 99% (95% CI, 94–100%) [18]. Invalid results were excluded. Laboratory data included two possible outcomes: 1) not currently infected (HCV RNA not detectable), and 2) current infection (HCV RNA detectable).

### 2.4. Self-Reported HCV Status

Participants self-completed a questionnaire assessing demographic, behavioural, and HCV infection status. Self-reported current HCV infection was assessed by multiple questions. History of HCV testing was obtained by combining answers given to: “Have you ever received an antibody test for HCV?” and “Have you ever received an RNA test for HCV?”. Among those who had ever been tested (antibody or RNA), participants were asked, “Have you ever been told you have HCV infection?”. If a participant had self-reported HCV diagnosis, they were asked “What is your current HCV status?”. Self-reported HCV status comprised four possible categories: (1) never tested, (2) tested, unknown status, (3) not infected, and (4) infected.

### 2.5. Outcomes

Outcomes were reported among all people with a valid HCV test result. The primary outcome was HCV status awareness defined as the proportion of participants whose HCV RNA test results and self-reported results were concordant (denominator comprised of all participants with HCV RNA test results). The secondary outcome was agreement, defined as the proportion of participants whose HCV RNA test results and self-reported results were concordant (denominator restricted to participants who self-reported HCV status).

In the whole study sample, the study assessed preferences and acceptability of a number of aspects of testing: same day results, time to receipt of results, delivery of results, modality of blood sampling, site of treatment, duration of treatment.

### 2.6. Study Oversight

All participants provided written informed consent before study procedures. The study protocol was approved by St. Vincent’s Hospital, Sydney Human Research Ethics Committee (30 July 2018, Reference number: HREC/18/SVH/101, primary study committee) and was conducted according to the Declaration of Helsinki and International Conference on Harmonization Good Clinical Practice (ICH/GCP) guidelines. The study was registered with clinicaltrials.gov registry (NCT03492112).

## 3. Results

### 3.1. Participant Characteristics

Overall, 101 participants were enrolled. The median age was 43 years, 31% (31 of 101) were women. At enrolment, 47% (47 of 101) injected drugs daily and 27% (27 of 101) were receiving OAT. The most commonly injected drugs in the previous month were methamphetamines (79%, 80 of 101) and heroin (55%, 56 of 101). Half of participants reported weekly attendance at an NSP in the last month (50%, 51 of 101) (Table 1). The majority of participants (99%, 100 of 101) had a valid HCV RNA test result. One person’s test produced an error and no valid RNA result was obtained.

### 3.2. Awareness of HCV Infection

Of 100 people with an HCV RNA result, 27% (27 of 100) had detectable HCV RNA (>limit of quantification). Overall, 65% (65 of 100) were aware of their HCV infection status, 10% (10 of 100) were incorrect about their infection status, and 25% (25 of 100) had either unknown testing results or had not been tested. Of 27 participants who were HCV RNA detectable, 48% (13 of 27) were aware of their HCV infection status (current HCV infection), 30% (8 of 27) incorrectly believed they were uninfected, and 22% (6 of 27) had either unknown testing results or had not been tested. Among the 73 participants who were HCV RNA undetectable, 71% (52 of 73) were aware of their infection status (not being infected), 3% (2 of 73) incorrectly believed they were infected and 26% (19 of 73) had either unknown testing results or had not been tested (Table 2). Among participants who self-reported previous HCV treatment (*n* = 26), 42% (11 of 26) were aware of their HCV status: 17% (1 of 6) of people previously treated with current HCV infection were aware of their status and 50% (10 of 20) of people previously treated without current HCV infection were aware of their status.

After excluding people who had never tested or had tested and did not know their status (*n* = 25), the agreement between self-reported HCV status and HCV RNA test results was 87% (65 out of 75). Among people with current HCV infection who self-reported their HCV status, agreement between self-reported status and HCV RNA test results was 62% (13 of 21). Among people with no current HCV infection who self-reported their HCV status, agreement between self-reported status and HCV RNA test results was 96% (52 of 54) and 4% (2 of 54) believed they had current HCV infection but did not (Table 2).

### 3.3. HCV Testing Acceptability

All participants were definitely or somewhat willing to receive HCV testing (100%, 101 of 101) or treatment (100%, 101 of 101) at an NSP. The majority of people would be willing to discuss their health with a peer NSP worker (78%, 79 of 101), or a nurse (88%, 87 of 101), and a smaller proportion were willing to discuss their health with other NSP workers (36%, 36 of 101) or a doctor (34%, 34 of 101).

Participants were asked to indicate how they would like to have their result delivered and who they would like to provide them with their result. The most acceptable result delivery method was in person (69%, 70 of 101), followed by text message (36%, 36 of 101), and by phone (28%, 28 of 101). The most frequently selected person to deliver the results was a peer NSP worker (87%, 88 of 101), followed by a nurse (81%, 82 of 101), other NSP worker (33%, 33 of 101), doctor (21%, 21 of 101), and other peer support worker (20%, 20 or 101).

Overall, 99% (100 of 101) indicated that fingerstick testing was very acceptable or somewhat acceptable. The one person that reported fingerstick testing being unacceptable gave the reason that it was painful (1%, 1 of 101). A lower proportion of participants (66%, 66 of 101) indicated that venepuncture blood testing was acceptable and 6% (6 of 101) indicated it was neither acceptable nor unacceptable. The main reasons for finding venepuncture blood testing somewhat or very unacceptable (29%, 29 of 101) were venous access difficulties (52%, 15 of 29), pain (24%, 7 of 29), time (10%, 3 of 29), and feeling the results will not be accurate (7%, 2 of 29).

When asked to choose between finger-stick testing and venepuncture testing, 90% (91 of 101) of participants preferred finger-stick testing. Among those that preferred finger-stick testing, the main reasons were speed (35%, 32 of 91), lack of pain (5%, 5 of 91), and the difficulties with the nurse performing venepuncture (44%, 40 of 91). Among those preferring venepuncture blood testing (10%, 10 of 101), the main reasons were speed (30%, 3 of 10), accuracy (20%, 2 of 10), and lack of pain (10%, 1 of 10).

The majority (95%, 96 of 101) indicated that they would prefer to receive same-day HCV test results, with 0% (0 of 101) preferring not to receive same-day results, and 5% (5 of 101) indicating that it did not matter. The main reasons for preferring same-day results included wanting it as soon as possible (18%, 17 of 96), less worry/stress (54%, 52 of 96), and convenience (26%, 25 of 96). For the multiple-choice question on time to receive result, the preferred option was the shortest time of 20 min (83%, 84 of 101), with 1% (1 of 101) indicating 30 min, 7% (7 of 101) one hour, and 6% (6 of 101) stating it does not matter (Supplementary Table S1). All participants were definitely or somewhat willing to receive treatment with either a 12-week regimen (100%, 101 of 101) or an 8-week regimen (100%, 101 of 101).

## 4. Discussion

In this sample of people who inject drugs attending a peer-based NSP in Sydney, Australia, one third of people were unaware of their current HCV status, despite the majority having previously tested for HCV. The study assessed a number of strategies that result in increased treatment initiation including rapid return of results, in-person delivery of results by peer NSP workers, and embedding HCV care in NSPs. These strategies are highly acceptable among this sample of people who inject drugs and are crucial to improve awareness of HCV status and access to treatment in this population.

Overall, 65% of participants were aware of their HCV status, comparable with other studies among people who inject drugs [19,20,21,22,23]. One study found that attending a GP or OAT clinic was associated with increased awareness of HCV status [22], highlighting the role of engagement with health services in increasing infection awareness. The lower awareness of HCV status among people who have current HCV infection (48%) emphasises the need for improved communication of results and supported follow-up to ensure people have the knowledge to seek treatment and adapt behaviours which have the potential for forward transmission. Clear communication will ensure people understand when they are being tested for HCV antibody vs. HCV RNA, and the implications of each result. Awareness was lower in the subsample of people with a history of HCV treatment (42%), supporting existing evidence on issues people face at different stages of the care cascade to understand their status [24]. A study in Australia found that 27% of people with unknown SVR12 had recently injected drugs, meaning they were not communicated their HCV status post-treatment [25]. Peer workers who have built good relationships with people attending the service are key actors to improve follow-up for testing and improve awareness of status.

There was 100% acceptability for people to be tested and treated for HCV in an NSP and high acceptability to discuss health with peer NSP workers (78%) and nurses (87%). Although this finding is promising, the study population consisted of people who consented to being tested and treated for HCV at the study site which may produce bias. All participants were recruited from a service staffed by a HCV peer support worker and nurse, indicating high acceptability for the model of care which participants were attending. The peer NSP worker in this service was an HCV specialist, possibly explaining the higher acceptability for receiving results compared to non-specialised peer workers. Discussing results with a doctor had low acceptability (34%), which could reflect the absence of doctors in the service which participants attend, but also reinforces the importance of integrating peer workers into the HCV care pathways in NSPs. As NSPs already serve a population with a high prevalence of HCV [26], they are well-positioned to support people to access testing, treatment, and post-cure follow-up to ensure they are aware of their status [24]. Given the frequency of reported NSP visits in this sample, delivering care in these settings is feasible and could improve treatment uptake [27]. This supports previous studies that demonstrated high acceptability [28] of co-locating HCV services in services frequently used by people who inject drugs.

The proportion of participants who preferred fingerstick blood sampling over venepuncture (90%) was higher than a previous study in drug treatment clinics from 2018, which found that 65% of participants preferred fingerstick over venepuncture [12]. Similar to the previous study [12], the drivers of preference for fingerstick blood sampling were venous access and speed of sampling. A number of environmental and behavioural factors can provoke venous damage in people who inject drugs [29], making the venepuncture process arduous and painful [30].

Importantly, 95% of participants wanted same-day results and half said same-day results would reduce stress. Stress may relate to one’s own health status but also around possibilities of transmission of HCV to other people. Fingerstick sample collection is likely to improve testing acceptability and uptake among people who inject drugs, facilitating improved treatment initiation [31]. There was no difference between acceptability of HCV treatment duration of eight weeks vs. 12 weeks, suggesting that treatment length is not a major factor influencing treatment uptake in this population.

There are several limitations to this study. The study was performed at a single NSP, staffed by a HCV peer support worker and nurse, and so results may not be generalizable to all populations of people who inject drugs nor all NSPs. Nevertheless, the sociodemographic characteristics of this study population are comparable to a 2022 national sentinel study of people who inject illicit drugs, recruited at NSPs and other harm reduction services in the capital cities of Australia [32] (similar on age, gender, homelessness, employment, recent use of heroin, recent use of methamphetamine). Furthermore, proportions of HCV status awareness align with a similar study in OAT clinics [22] indicating consistency across other settings in Australia. The self-administered questionnaire did not capture reasons for never testing nor further information on people who were tested but did not know their result. Further investigation on these issues would help inform improvements in testing and treatment pathways. When asked to self-report HCV status, there was no option to indicate if a person did not know if they had ever been tested for HCV. The measures of acceptability may be influenced by people’s experience at the site and may reflect satisfaction with the current model of care.

Two thirds of the overall study sample were aware of their HCV status and half of those with current HCV were aware of their status, evidencing the need for increased testing, communication of results, and post-treatment follow-up. Aspects of testing and treatment which serve to decentralise, simplify, and accelerate HCV care pathways are acceptable among this sample of people who inject drugs, indicating possibilities for improving awareness of HCV infection status and facilitating greater patient empowerment to engage in testing and treatment to advance HCV elimination globally.

## Figures and Tables

**Table 1:** Baseline characteristics.

Variable	Overall (*n* = 101)
Age, median years (IQR)	43 (37–47)
Women	31 (31%)
High school or higher education	38 (38%)
Homeless *	17 (17%)
Employment	
Full-time employment	2 (2%)
Part-time employment	5 (5%)
Government assistance	94 (93%)
Incarceration	
Recent	21 (21%)
Ever	50 (50%)
Never	30 (30%)
Injecting drug use in the previous month	
None	4 (4%)
Heroin	56 (55%)
Cocaine	13 (13%)
Methamphetamines	80 (79%)
Other opioids	31 (31%)
Injecting drug use frequency in the previous month	
Never	4 (4%)
<daily	50 (50%)
≥daily	47 (47%)
Attended needle syringe program in previous month	
No	8 (8%)
Yes, less than weekly	42 (42%)
Yes, more than weekly less than daily	44 (43%)
Yes, daily	7 (7%)
Hazardous alcohol use in the previous month	41 (41%)
Current OAT	
No	74 (73%)
Yes, methadone	18 (18%)
Yes, buprenorphine	9 (9%)

Data are *n* (%), or median (Interquartile Range). High school = completing 13 years of schooling. OAT = opioid agonist therapy. * Homelessness was defined as spending majority of nights in the last month in no usual residence, a shelter or squat.

**Table 2:** Self-reported HCV infection status and actual HCV infection status among people with HCV test result (*n* = 100).

		Self-Reported Current HCV Status				Total
		Never Tested	Tested, Unknown	Not Infected	Infected	
		*n* (% row)	*n* (% row)	*n* (% row)	*n* (% row)	*n*
HCV result at enrolment	No current HCV infection (HCV RNA not quantifiable)	2 (3%)	17 (23%)	52 (71%)	2 (3%)	73
	Current HCV infection (HCV RNA quantifiable)	1 (4%)	5 (19%)	8 (30%)	13 (48%)	27
	Total	3 (3%)	22 (22%)	60 (60%)	15 (15%)	100

## Data Availability

The individual deidentified participant data (including data dictionaries) that support the findings of this study (text, tables, figures, and appendices) are available from the corresponding author upon reasonable request. The study protocol is included as Supplementary Material and the study is registered at ClinicalTrials.gov: NCT03492112. Data may be requested from the corresponding author by email (with an appropriate plan for the use of data) by investigators to perform individual-level meta-analyses that has been approved by an independent review committee identified for this purpose. Proposals may be submitted immediately following publication.

## Supplementary Materials

The following supporting information can be downloaded at: https://www.mdpi.com/article/10.3390/v14112463/s1, Table S1: Acceptability and preferences for aspects of HCV testing and treatment (*n* = 101).
