# Supplementary material for: Awareness of HCV Status and Preferences for Testing and Treatment among People with Recent Injecting Drug Use at a Peer-Led Needle and Syringe Program: The TEMPO Pilot Study
Source: Viruses. 2022 Nov 7;14(11):2463. doi: 10.3390/v14112463 (PMC9696805; doi:10.3390/v14112463)
Supplement: Supplementary file 1 [file viruses-14-02463-s001.zip › viruses-1959382-supplementary.pdf]

Table S1. Acceptability and preferences for aspects of HCV testing and treatment (n = 101).

Variable	Overall
**Total**	101
**Would you prefer to get your HCV results on the same day?, n = 101 (%)**	
Yes	96 (95%)
No	0 (0%)
Doesn't matter	5 (5%)
**What is the main reason you want your result on the same day?, n = 96 (% of those who want same day results)**	
I want them as soon as possible	17 (18%)
Less worry/stress	52 (54%)
More convenient	25 (26%)
Other	2 (2%)
**Preferred time to receive HCV test results, n = 101 (%)**	
Within 20 minutes	84 (83%)
Within 30 minutes	1 (1%)
Within one hour	7 (7%)
Within two hours	2 (2%)
After 2 hours, but on the same day	0 (0%)
After today, but within one week	1 (1%)
Does not matter	6 (6%)
**For results within an hour, preferred method of result provision, multiple responses possible, n = 101 (%)**	
Text message	36 (36%)
By phone	28 (28%)
In person	70 (69%)
From my GP	9 (9%)
From my OAT clinic	5 (5%)
**For results within an hour, preferred person to provide the result, multiple responses possible, n = 101 (%)**	
Nurse	82 (81%)
Peer needle and syringe program worker	88 (87%)
Other needle and syringe program worker	33 (33%)
Peer support worker	20 (20%)
Doctor	21 (21%)
Other	4 (4%)
**Acceptability of fingerstick blood testing, n = 101 (%)**	
Very acceptable	93 (92%)
Somewhat acceptable	7 (7%)
Neither acceptable nor unacceptable	0 (0%)
Somewhat unacceptable	1 (1%)
Not at all acceptable	0 (0%)
**Reason for finding fingerstick blood testing unacceptable, n = 1 (% of those who found fingerstick unacceptable)**	
It takes too much time	0 (0%)
I feel the results will not be accurate	0 (0%)
It is painful	1 (100%)
**Acceptability of venepuncture blood testing, n = 101 (%)**	
Very acceptable	41 (41%)
Somewhat acceptable	25 (25%)
Neither acceptable nor unacceptable	6 (6%)

Somewhat unacceptable	22 (22%)
Not at all acceptable	7 (7%)
**Reason for finding venepuncture blood testing unacceptable, *n* = 29 (% of those who found finger-stick unacceptable)**	
It takes too much time	3 (10%)
I feel the results will not be accurate	2 (7%)
I have poor veins and it is difficult for the nurse or doctor to get blood from me	15 (52%)
It is painful	7 (24%)
Other	2 (7%)
**Preferred testing method, *n* = 101 (%)**	
Venepuncture	10 (10%)
Fingerstick test	91 (90%)
**Reason for preferring venepuncture, *n* = 10 (% of those that prefer venepuncture)**	
It is quick	3 (30%)
I know it will be accurate	2 (20%)
I am used to having testing done this way	0 (0%)
It does not hurt	1 (10%)
Other	4 (40%)
**Reason for preferring fingerstick testing, *n* = 91 (% of those that prefer fingerstick)**	
It is quick	32 (35%)
I know it will be accurate	0 (0%)
It does not hurt	5 (5%)
The nurse usually has trouble taking my blood	40 (44%)
Other	14 (15%)
**Needle and syringe program attendance, *n* = 101 (%)**	
Not in the last month	8 (8%)
Less than weekly	42 (42%)
Weekly	28 (28%)
A few times a week	16 (16%)
Daily	7 (7%)
**Willingness for testing at a needle and syringe program, *n* = 101 (%)**	
Definitely willing	97 (96%)
Somewhat willing	4 (4%)
Neither willing nor unwilling	0 (0%)
Somewhat unwilling	0 (0%)
Not at all willing	0 (0%)
**Willingness for HCV treatment at a needle and syringe program, *n* = 101 (%)**	
Definitely willing	96 (95%)
Somewhat willing	5 (5%)
Neither willing nor unwilling	0 (0%)
Somewhat unwilling	0 (0%)
Not at all willing	0 (0%)
**Willingness for 12 week HCV treatment, *n* = 101 (%)**	
Definitely willing	96 (95%)
Somewhat willing	5 (5%)
Neither willing nor unwilling	0 (0%)
Somewhat unwilling	0 (0%)
Not at all willing	0 (0%)
**Willingness for 8 week HCV treatment, *n* = 101 (%)**	
Definitely willing	96 (95%)
Somewhat willing	5 (5%)
Neither willing nor unwilling	0 (0%)
Somewhat unwilling	0 (0%)

Not at all willing	0 (0%)
**Who would you be willing to discuss your health with at a needle and syringe program? Multiple responses possible, *n* = 101 (%)**	
Peer needle and syringe program worker	79 (78%)
Other needle and syringe program workers	36 (36%)
Nurses	88 (87%)
Doctors	34 (34%)
